# The clinical and bioinformatics analysis for the role of antihypertension drugs on mortality among patients with hypertension hospitalized with COVID‐19

**DOI:** 10.1002/jmv.27914

**Published:** 2022-06-16

**Authors:** Liyang Zhao, Yusi Li, Wenjuan Yi, Kuo Yan, Chao Yang, Sridhar Radhakrishnan, Rui Li, Ruirong Tan, Gang Fan, Mengyuan Dai, Miao Liu, Ning‐Yi Shao

**Affiliations:** ^1^ Department of Biomedical Sciences, Faculty of Health Sciences University of Macau Taipa, Macau China; ^2^ Department of Dermatology Zhongnan Hospital of Wuhan University Wuhan China; ^3^ Institute of Cell and Neurobiology Charité Medical University Berlin Germany; ^4^ Department of Medicine Brigham and Women's Hospital, Harvard Medical School Boston Massachusetts USA; ^5^ Cancer Science Institute of Singapore National University of Singapore Singapore Singapore; ^6^ Department of Radiation Oncology, Sichuan Cancer Hospital & Institute, Sichuan Cancer Center, School of Medicine University of Electronic Science and Technology of China Chengdu China; ^7^ Translational Chinese Medicine Key Laboratory of Sichuan Province Sichuan Institute for Translational Chinese Medicine, Sichuan Academy of Chinese Medicine Sciences Chengdu China; ^8^ Department of Urology Huazhong University of Science and Technology Union Shenzhen Hospital, The 6th Affiliated Hospital of Shenzhen University Health Science Center Shenzhen China; ^9^ Department of Gynecological Oncology Zhongnan Hospital of Wuhan University Wuhan China; ^10^ Department of Pathology Brigham and Women's Hospital, Harvard Medical School Boston Massachusetts USA; ^11^ MoE Frontiers Science Center for Precision Oncology University of Macau Taipa, Macau SAR China

**Keywords:** SARS coronavirus, Virus classification, Infectious bronchitis virus, Virus classification, Biostatistics & Bioinformatics

## Abstract

Comorbidities such as hypertension could exacerbate symptoms of coronaviral disease 2019 (COVID)‐19 infection. Patients with hypertension may receive both anti‐COVID‐19 and antihypertension therapies when infected with COVID‐19. However, it is not clear how different classes of anti‐hypertension drugs impact the outcome of COVID‐19 treatment. Herein, we explore the association between the inpatient use of different classes of anti‐hypertension drugs and mortality among patients with hypertension hospitalized with COVID‐19. We totally collected data from 278 patients with hypertension diagnosed with COVID‐19 admitted to hospitals in Wuhan from February 1 to April 1, 2020. A retrospective study was conducted and single‐cell RNA‐sequencing (RNA‐Seq) analysis of treatment‐related genes was performed. The results showed that Angiotensin II receptor blocker (ARB) and calcium channel blocker (CCB) drugs significantly increased the survival rate but the use of angiotensin‐converting enzyme inhibitor/β‐block/diuretic drugs did not affect the mortality caused by COVID‐19. Based on the analysis of four public data sets of single‐cell RNA‐Seq on COVID‐19 patients, we concluded that *JUN*, *LST1* genes may play a role in the effect of ARB on COVID‐19‐related mortality, whereas *CALM1* gene may contribute to the effect of CCB on COVID‐19‐related mortality. Our results provide guidance on the selection of antihypertension drugs for hypertensive patients infected with COVID‐19.

## 1 INTRODUCTION

The global pandemic coronaviral disease 2019 (COVID‐19) caused by severe acute respiratory syndrome coronavirus 2 (SARS‐CoV‐2), one of the most widely transmissible RNA viruses ever, keeps impacting millions of patients.^1^ Although a wide range of vaccines and drugs against the viruses have been developed, multiple viral mutants with increasing infectivity and transmissibility, such as Delta and Omicron, are emerging consecutively.^2^ Eradicating COVID‐19 becomes an everlasting battle for the entire human society.^3^ The patients' responses to SARS‐CoV‐2 infection vary with their own conditions, such as age, gender, obesity and comorbidities. In particular, comorbidities in the COVID‐19 situation frequently bring about the aggravation of COVID‐19 symptoms and they thus have to be treated with combined therapeutic strategies for both COVID‐19 and the comorbidity. However, the reciprocal effects between COVID‐19 and conventional disease medicament remain to be analyzed.^4^

Hypertension is one of the most fatal diseases and threatens more than 100 million people in the last three decades worldwide.^5^ It has become one of the common comorbidity with COVID‐19. It has been reported that COVID‐19 patients with hypertension, who suffer from a higher risk of death, have to consequently administer drugs for hypertension and COVID‐19 simultaneously. However, one recent report demonstrates that angiotensin‐converting enzyme inhibitors (ACEI) and Angiotensin II receptor blocker (ARB) may reduce the mortality risk of COVID‐19 patients, raising the question if and how hypertension drugs interfere with virus replication in COVID‐19 patients. Additionally, another concern is how the expression of genes associated with hypertension drugs is affected by SARS‐CoV‐2 infection, particularly in patients bearing both hypertension and COVID‐19.^6^

To protect these patients' health, it is worthwhile to study the effects of existing hypertension medicine upon COVID‐19 pathology. Here we analyzed the age‐ and sex‐dependent effects of diverse hypertension drugs on COVID‐19 symptoms and found that calcium channel blockers (CCBs) played a protective role in the progression of COVID‐19 pathogenesis in a statistically significant manner, particularly in the patients over 70 years old. Furthermore, we first investigated the expression patterns of the hypertension drug‐related genes in COVID‐19 patients by data set mining and modeling, suggesting the molecules being potentially involved in both hypertension and COVID‐19 pathology, such as JUN, LST1, and SLC18A2. Our study therefore adds essential medicinal information to COVID‐19 protection.

## 2 METHODS

### 2.1 Data collection

This was a retrospective study. Data of 717 hospitalized patients with COVID‐19 were obtained from Wuhan's government‐designated hospitals for the treatment of COVID‐19. COVID‐19 was diagnosed based on the New Coronavirus Pneumonia Prevention and Control Program published by the National Health Commission of China. We selected 278 hypertension patients from all these cases with COVID‐19 and hypertension was diagnosed as systolic blood pressure ≥ 140 mmHg and/or diastolic blood pressure ≥ 90 mmHg three times on different days without taking antihypertensive drugs.^7^ Patient demographics (age and gender), time of admission, time of discharge, therapeutic interventions of hypertension, and clinical outcomes during the hospitalization were collected from the e‐health system. Hypertension treatment drugs for patients included ACEI, ARB, CCB, β‐blockers, and diuretic. Recruited patients who received combination medication were considered to receive each drug in our study, separately. The following inclusion and exclusion criteria were used to identify patients in this study. The inclusion criteria were hypertensive patients diagnosed as COVID‐19 aged above 35 years old, who were admitted to the hospitals mentioned above from February 1 to April 1, 2020. Patients without completed medical records were excluded (cured during the follow‐up period but the cured time was not recorded/hospitalized during the follow‐up period, but it was not known whether an outcome event occurred). Informed consent was obtained from included patients or their representatives, and the study was approved by the participating hospitals' review boards.

## 3 STATISTICAL ANALYSIS

A descriptive analysis was used to describe the characteristics of included patients. The primary endpoint was 75‐day all‐cause death. All eligible patients were included in the analysis of 75‐day all‐cause death. The Kaplan‐Meier method was performed to display the survival probability and the differences in survival were analyzed by the log‐rank test. A univariate Cox proportional‐hazards model was conducted to have a primary analysis of the relationship between each independent variable and outcome, and statistically significant factors were taken into the multivariable analysis. Multivariable analyses with the Cox proportional hazards model were used to estimate the adjusted hazard ratio (HR) between in‐hospital use of different drug therapy and all‐cause mortality in patients with hypertension and hospitalized due to COVID‐19. Furthermore, the multivariate Cox proportional hazards model was used for subgroup analysis for different sex groups and age groups, respectively, to explore the differences in the effects of various drug therapy in the subgroups. 70 was the age cutoff. All data analyses were conducted by R studio 3.6.2. The “survival” and “survminer” packages were used for the survival analysis and Cox regression analysis. All statistical tests were two‐sided with a statistically significant *p*‐value < 0.05.

### 3.1 Single‐cell RNA‐sequencing (RNA‐Seq) analysis

We downloaded four data sets from three publications and the downloaded data have been aligned and annotated; the R package Seurat V3 was used for filtering, standardization, conversion, dimensionality reduction, clustering, gene differential expression analysis, and visualization. The standards of filtering, clustering, and dimensionality reduction were consistent with the respective original texts. “MAST” was used for gene differential expression analysis.

The bronchoalveolar lavage fluid (BALF) single‐cell RNA‐Seq raw data from Liao et al.^8^ including three moderate, six severe cases, and three healthy controls were downloaded from the Gene Expression Omnibus (GEO) database (accession number GSE145926). In addition, there was also a case of health control data from GEO GSM3660650. According to the original text, 66 452 cells were obtained by using the following parameters: nFeature > 200 and <6000, nCount > 1000, mitochondrial gene percentage < 10. The function “Lognormalize” was used for normalization. Then, we identified the top 2000 highly variable genes by “VST” method in the function FindVariableFeatures. The filtered cells were integrated to removing the batch effect (dim=1: 50). In ScaleData, “nCount_ RNA” and “percent. mito” has been regressed. According to the cell source, the cluster was divided into three clusters: healthy, mild, and severe. Finally, the significant genes with adjusted *p* < 0.05 in different groups were obtained for downstream analysis by performing gene differential expression analysis with “MAST” in FindAllMarkers function.

The peripheral blood mononuclear cells single‐cell RNA‐Seq data from Wilk et al.^9^ including seven severe cases and six healthy controls were downloaded from the GEO database (accession number GSE150728). According to the following parameters, we filtered, normalized, and integrated the data of 14 experimental subjects (1 patient was sampled once before and after intubation): 1000 < nCount < 15 000, mitochondrial genes < 20, at least 10 genes must be expressed in each cell. Then the rest of the analyses followed the analysis of GSE145926 data set.

The single‐cell RNA‐Seq data of 27 cases of nasopharyngeal or pooled nasopharyngeal/pharyngeal swabs (NSs; 14 cases of moderate, 13 cases of critical), 2 cases of bronchial lavages (BLs; critical), and 5 cases of control from Chua et al.^12^ were downloaded from doi:10.6084/m9.figshare.12436517.10. To be as consistent as possible with the original text, we used the preprocessed data uploaded by the original author. On this basis, we identified the cluster as control and critical (BLs), and control, moderate, and critical (NSs), according to the source of cell sampling. Finally, the significant genes with adjusted *p* < 0.05 in different groups were obtained for downstream analysis by performing gene differential expression analysis with “MAST” in FindAllMarkers function.

## 4 RESULTS

A total of 278 patients were selected in the study (Table 1). The mean age of diagnosis was 66.5 years (range from 35 to 97 years). One hundred and thirty five (48.56%) were female and 143 (51.44%) were male. CCB was the most commonly used antihypertensive drug, followed by ARB, ACEI, β‐blockers, and diuretics with 167, 72, 31, 17, and 9 patient users, respectively. Forty‐seven patients did not receive any of the above drugs. During a 75‐day follow‐up duration, a total of 35 deaths (12.59%) occurred among 278 patients. Among the total 35 deaths, 6 deaths were treated with 2 drugs and only 1 drug was received by 29 other deaths. The number of deaths in patients receiving ACEI, ARB, CCB, β‐blocker, diuretic drugs, and not receiving any antihypertensive drugs was 3, 3, 12, 2, 0, 21, respectively.

**Table 1:** Characteristics of the study sample, *n* = 278

Variable	Number (*n*)	Percent (%)	Number of deaths	Percent (%)
Sex				
Female	135	48.56	12	8.89
Male	143	51.44	23	16.08
Age (years)				
<70	169	60.79	13	7.69
≥70	109	39.21	22	20.18
Therapy				
ACEI	31	11.15	3	9.68
ARB	72	25.90	3	4.17
CCB	167	60.07	12	7.19
β‐blockers	17	6.12	2	11.76
Diuretic	9	3.24	0	0
Nontherapy	47	16.90	21	44.68
Outcomes				
Survived	243	87.41	–	–
Death	35	12.59	–	–

Abbreviations: ACEI, angiotensin‐converting‐enzyme inhibitors; ARB, Angiotensin II receptor blockers; CCB, calcium channel blockers.

**Figure 1:**
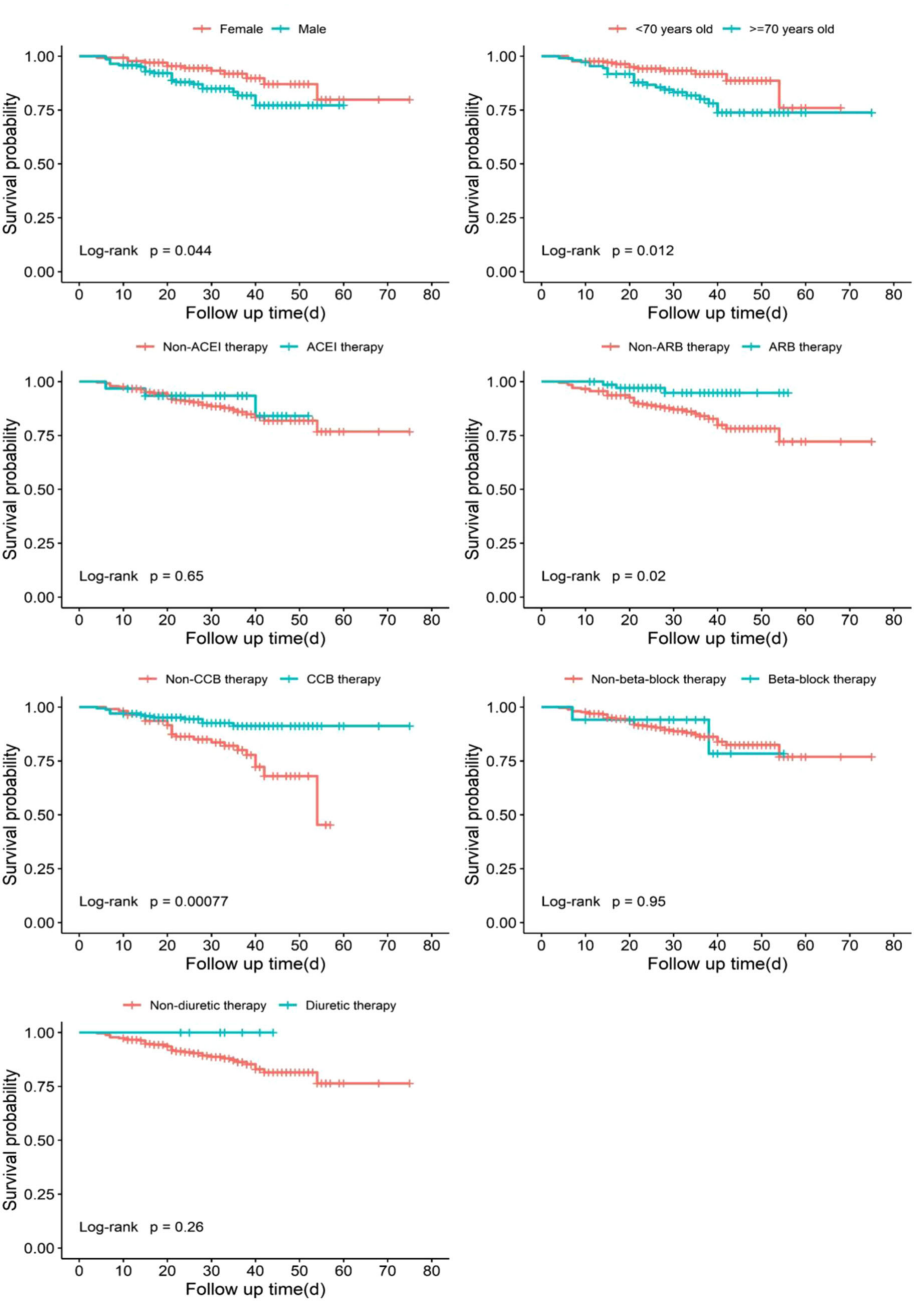
Kaplan–Meier curve by sexes, age groups, and different therapy. *p* < 0.05 was statistically significant.

Survival analysis (Figure 1) showed that the survival rate of hypertensive patients infected with SARS‐COV‐2 was significantly correlated with sex, age, and certain types of antihypertensive drugs, respectively. Compared with females, survival was significantly lower in male patients (*p* = 0.04). The survival rate of patients equaled to or over 70 years old was significantly lower than that of patients under 70 years old (*p* = 0.01). Intriguingly, the survival rate of patients who did not receive ARB was significantly lower than that of patients who received ARB (*p* = 0.02). Similarly, the survival rate of patients who did not receive CCB was significantly lower than that of patients who received CCB (*p* < 0.01). Notably, the survival rate at day 54 was 45.3%, which was <50%, in the group not receiving CCB. Although the survival rate in the follow‐up period was higher than 50% in the group receiving CCB. The survival rate of hypertensive patients with COVID‐19 was not significantly associated with whether receiving ACEI/β‐block/diuretic. Taken together, hypertensive patients taking ARB or CCB and infected with COVID‐19 had a higher survival rate from all‐cause death.

The adjusted HR for the association of included variables for all‐cause mortality in patients with hypertension and hospitalized due to COVID‐19 were demonstrated in Table 2. The detected risk for all‐cause mortality was higher in patients equal to or above 70 years old compared with patients aged below 70 (adjusted HR, 2.24 [95% confidence interval {CI}, 1.12, 4.49]). Patients who received ARB had a lower risk of all‐cause mortality than those who did not receive ARB (adjusted HR, 0.22 [95% CI, 0.07, 0.74]). Regarding CCB, patients who received CCB witnessed a lower risk of all‐cause mortality versus those who did not have CCB (adjusted HR, 0.23 [95% CI, 0.11, 0.47]). The results of subgroup analysis were demonstrated in Tables 3 and 4. The female patients who received CCB had a lower risk of all‐cause mortality than those who did not receive CCB (adjusted HR, 0.10 [95% CI, 0.02, 0.45]), whereas no significant difference was found among the male patients. Moreover, CCB intake was associated with a significantly lower risk of all‐cause mortality among patients aged ≥70 years (adjusted HR, 0.12 [95% CI, 0.05, 0.34]), but no significant association was found in patients aged <70 years.

**Table 2:** Cox proportional hazards model

	Univariate		Multivariate	
	HR (95% CI)	*p*	HR (95% CI)	*p*
Sex				
Female	1		1	
Male	2.03 (1.01, 4.09)	0.048*	1.74 (0.86, 3.55)	0.13
Age (years)				
<70	1		1	
≥70	2.35 (1.18, 4.68)	0.01*	2.24 (1.12, 4.49)	0.02*
Therapy				
ACEI	0.76 (0.23, 2.49)	0.65		
ARB	0.27 (0.08, 0.88)	0.03*	0.22 (0.07, 0.74)	0.02*
CCB	0.32 (0.16, 0.64)	<0.01*	0.23 (0.11, 0.47)	<0.01*
β‐blockers	0.95 (0.23, 3.97)	0.94		
Diuretic	NA	NA		

Abbreviations: ACEI, angiotensin‐converting‐enzyme inhibitors; ARB, Angiotensin II receptor blockers; CCB, calcium channel blockers; CI, confidence interval; HR, hazard ratio; NA, not available.

* *p* < 0.05.

**Table 3:** Cox proportional hazards model among sexes

	Female		Male	
Therapy	HR (95% CI)	*p*	HR (95% CI)	*p*
Age	3.12 (0.93, 10.40)	0.06	2.37 (0.97, 5.76)	0.06
ARB	NA	NA	0.59 (0.16, 2.22)	0.44
CCB	0.10 (0.02, 0.45)	<0.01*	0.44 (0.19, 1.03)	0.06

Abbreviations: ARB, Angiotensin II receptor blockers; CCB, calcium channel blockers; CI, confidence interval; HR, hazard ratio; NA, not available.

* *p* < 0.05.

**Table 4:** Cox proportional hazards model among different age groups

	Age <70 years		Age ≥70 years	
Therapy	HR (95% CI)	*p*	HR (95% CI)	*p*
Sex	2.42 (0.71, 8.26)	0.16	1.37 (0.56, 3.34)	0.50
ARB	0.37 (0.08, 1.83)	0.23	0.16 (0.02, 1.23)	0.08
CCB	0.63 (0.20, 1.97)	0.43	0.12 (0.05, 0.34)	<0.01*

Abbreviations: ARB, Angiotensin II receptor blockers; CCB, calcium channel blockers; CI, confidence interval; HR, hazard ratio; NA, not available.

* *p* < 0.05.

**Figure 2:**
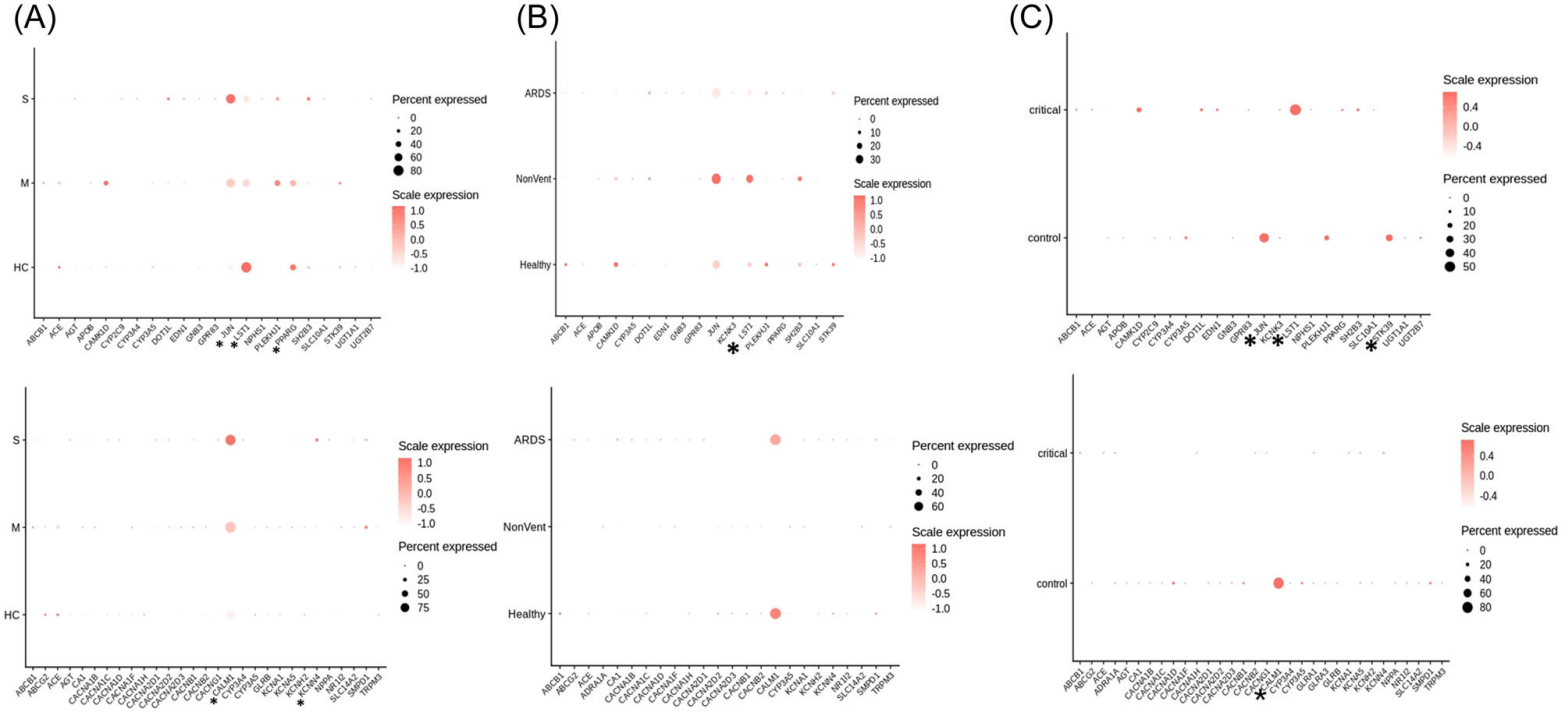
Heatmaps of the expression levels of genes associated with the hypertension treatments in bronchoalveolar lavage fluid (BALF) of coronaviral disease 2019 (COVID‐19) patients. *p < 0.05. ARDS, ventilated patients with acute respiratory distress syndrome; HC, healthy controls; M, moderate patients; S, severe; NoVent, patients without ventilation; Healthy, healthy control; critical, severe; control, healthy control. (A) The expression patterns of Angiotensin II receptor blocker (ARB; upper)‐ and calcium channel blocker (CCB; lower)‐associated genes in BALF. (B) the expression patterns of ARB (upper) and CCB (lower)‐associated genes in peripheral blood. (C) The expression patterns of ARB (upper)‐ and CCB (lower)‐associated genes in nasopharyngeal/pharyngeal swabs.

To investigate the molecular mechanisms underlying the effect of hypertension drugs on the survival rate of COVID‐19 patients, we searched the drug‐gene databases including Broad Institute's CMAP,^10^ Stanford's PharmGKB,^11^ and curate various hypertension drug‐associated gene lists. Further, we downloaded and analyzed the single‐cell RNA‐seq data sets of COVID‐19 patients from three different sources and examined the expression patterns of hypertension drug‐associated genes. The gene expression profiles were extracted from the BALF,^8^ peripheral blood,^9^ and NS/pharyngeal swabs.^12^ We found that although the transcriptome data sets were independent, the differential expressed genes related with ARB and CCB show similar patterns between the severe versus control comparisons in the COVID‐19 patients (Figure 2). JUN, LST1, and CAML1 expression were increased in COVID‐19 patients.

## 5 DISCUSSION

The highly contagious SARS‐CoV‐2 virus endangers human health in many aspects and has been found to impose its effects on the pathogenesis of other diseases, such as cancer, diabetes, and hypertension.^6^, ^13^, ^14^, ^15^ On the other hand, current commercially available drugs against hypertension may intervene with COVID‐19 pathological consequences, indicated by the recent report that angiotensin‐associated hypertension drugs, ACEI and ARB, may curtail the risk of patients' death caused by SARS‐CoV‐2 infection.^6^ However, the pathophysiological effects of interaction between COVID‐19 and other categories of hypertension medicine are yet unclear. To address this issue, we performed systematic analysis for a wide range of hypertension drugs on their effects upon COVID‐19 pathogenesis. Our results depict that COVID‐19 patients with hypertension had a mortality rate of 12.59%, which was much higher than COVID‐19 patients without hypertension (3.42%, Table S1). Hypertension drugs behave variably on mortality hazards and exhibit sex‐ or age‐dependent activities on COVID‐19 patients. The nontherapy group demonstrated a mortality rate of 44.68%, which is much higher than the treated groups with a mortality rate of between 0% and 11.76%, suggesting the very necessity of medicine administration for hypertension patients infected by SARS‐CoV‐2 virus. CCB drugs elicit protective effects for both male and female COVID‐19 patients, particularly for those at the age of over 70 years old, supported by the observation that CCB drugs lower the overall mortality rate in a statistically consistent fashion. Our data provide advisory suggestions about concerning to clinical drug classification by sex and ag for hypertension patients carrying SARS‐CoV‐2.

The hypertension medicaments alleviate patients' symptoms via different mechanisms. ACEI and ARB broaden the veins and arteries to reduce blood pressure by preventing the action of Angiotensin II, a chemical that constricts the blood vessels. CCB are a group of calcium antagonists against the ion movement into the heart or artery cells to assist heart pumping. The finding that hypertension drugs act differentially on COVID‐19 patients may be due to their distinct biochemical functions.

To further dissect the molecular mechanisms underlying the interactive responses between hypertension drugs and SARS‐CoV‐2 virus, we next investigate transcriptomic profiling of the genes related to these drugs in COVID‐19 patients by data mining and mathematical modeling. Our analysis suggests that the expression patterns of hypertension drug‐related genes are indeed altered in COVID‐19 patients. The expression levels of two ARB associated genes, JUN and LST1, have been found substantially increased in COVID‐19 patients relative to controls, providing a supportive interpretation for the previous report.^6^ Although the etiology of hypertension is unknown, there is evidence that the nature of hypertension is chronic inflammation. Changes in inflammatory factors, immune cell ratios, and phenotypes can be observed in hypertensive patients.^16^ JUN and LST1 are associated with inflammation and immunity.^17^ JUN is a target gene of the antihypertensive drug Irbesartan and LST1 affects leukocyte abundance and T‐cell proliferation.^17^, ^18^ CALM1 is a target gene of the antihypertensive drug Felodipine, which regulates calcium channels.^19^ Therefore, the use of ARB or CCB treatment may decrease the expression level of related genes in infected patients, thereby improving the survival rate of COVID‐19 hypertensive patients.^16^, ^17^, ^18^, ^19^ The question if the expression of these genes is directly regulated by SARS‐CoV‐2 infection still needs to be further explored. There were a few limitations in our study. Firstly, the number of included patients treated with diuretic/β‐blockers was small. Second, we did not consider the effect of interaction between combination medications. However, our analysis for the first time describes the links between hypertension drugs, disease‐related genes and COVID‐19 pathology, and more importantly, delivers the central information that some medicine on market may influence patients' reactions in comorbidity to SARS‐CoV‐2 infection through modulating expression profiles of disease‐related genes. Additionally, our research highlights the clinical consideration of the interaction between commercial medicine and the SARS‐CoV‐2 virus, particularly in the patients with comorbidity.

## AUTHOR CONTRIBUTION

Miao Liu, Mengyuan Dai, and Ning‐Yi Shao designed the study and contributed to data interpretation. Wenjuan Yi, Mengyuan Dai, and Miao Liu contributed to data collection. Ning‐Yi Shao, Liyang Zhao, and Yusi Li compiled and analyzed the data. Miao Liu, Ning‐Yi Shao, Kuo Yan, and Chao Yang wrote the manuscript. Miao Liu and Ning‐Yi Shao reviewed and edited the manuscript. Miao Liu, Sridhar Radhakrishnan, Rui Li, Ruirong Tan, Mengyuan Dai, and Gang Fan provided the technical and material support. All authors reviewed the manuscript and edited it for intellectual content, and gave final approval for this version to be published.

## CONFLICT OF INTEREST

The authors declare no conflict of interest.

## Supporting information

Supporting information.

## Data Availability

The data that support the findings of this study are available on request from the corresponding author.
