# Supplementary material for: The clinical and bioinformatics analysis for the role of antihypertension drugs on mortality among patients with hypertension hospitalized with COVID‐19
Source: J Med Virol. 2022 Jun 16;94(10):4727–34. doi: 10.1002/jmv.27914 (PMC9347732; doi:10.1002/jmv.27914)
Supplement: Supplementary file 1 — Supporting information. [file JMV-94-4727-s001.docx]

Table S1 Characteristics of the COVID-19 patients without hypertension, n=439

| Variable | Number (n) | Percent (%) | Number of deaths | Percent (%) |
| --- | --- | --- | --- | --- |
| **Sex** |  |  |  |  |
| Female | 231 | 52.62 | 4 | 1.73 |
| Male | 208 | 47.38 | 11 | 5.29 |
| **Age** |  |  |  |  |
| <70 | 357 | 81.32 | 7 | 1.96 |
| >=70 | 82 | 18.68 | 8 | 9.76 |
| **Outcomes** |  |  |  |  |
| Survived | 424 | 96.58 | - | - |
| Death | 15 | 3.42 | - | - |
